# Stigma, Social Cohesion, and HIV Risk Among Sexual and Gender Minorities in Two Cities in Zimbabwe

**DOI:** 10.1007/s10461-022-03622-8

**Published:** 2022-03-18

**Authors:** Sophia S. Miller, Joanne E. Mantell, Lauren E. Parmley, Godfrey Musuka, Innocent Chingombe, Munyaradzi Mapingure, John H. Rogers, Yingfeng Wu, Avi J. Hakim, Owen Mugurungi, Chesterfield Samba, Tiffany G. Harris

**Affiliations:** 1 ICAP at Columbia University, New York, NY USA; 2 HIV Center for Clinical and Behavioral Studies, Department of Psychiatry, Division of Gender, Sexuality and Health, New York State Psychiatric Institute and Columbia University Irving Medical Center, New York, NY USA; 3 Division of Global HIV and TB, Center for Global Health, U.S. Centers for Disease Control and Prevention, Harare, Zimbabwe; 4 Division of Global HIV and TB, Center for Global Health, U.S. Centers for Disease Control and Prevention, Atlanta, GA USA; 5 AIDS and TB Programme, Zimbabwe Ministry of Health and Child Care, Harare, Zimbabwe; 6 GALZ, Harare, Zimbabwe; 7 Department of Epidemiology, Mailman School of Public Health, Columbia University, New York, NY USA

**Keywords:** Stigma, Social cohesion, HIV, MSM, Sub-Saharan Africa

## Abstract

Though stigma is a recognized contributor to the disproportionate HIV burden among sexual and gender minorities (SGM) in sub-Saharan Africa, data describing this association among Zimbabwean SGM are limited. We examined relationships between SGM stigma and HIV and the potential for social cohesion to moderate the association among Zimbabwean men who have sex with men, transgender women, and genderqueer individuals. Consenting participants (n = 1511) recruited through respondent-driven sampling for a biobehavioral survey in Harare and Bulawayo completed structured interviews and received HIV testing. Reported SGM stigma was common (68.9% in Harare and 65.3% in Bulawayo) and associated with HIV infection in Harare (adjusted prevalence ratio [aPR] = 1.82, 95% confidence interval [CI] = 1.27–2.62) and Bulawayo (aPR = 1.51, 95% CI = 1.15–2.00) in relative risk regression. Social cohesion did not moderate these relationships. Findings demonstrate stigma’s association with HIV vulnerability among Zimbabwean SGM, highlighting the need for stigma-mitigation to reduce HIV transmission in this population.

## Introduction

In sub-Saharan Africa (SSA), men who have sex with men (MSM) are disproportionally affected by HIV, even in countries with generalized epidemics [1–3]. A 2019 meta-analysis demonstrated a five-fold increase in average HIV prevalence among MSM compared to that of SSA’s general adult male population, with as high as 27- and 43-fold increases reported in Ghana and Senegal, respectively [1]. Although there are limited data describing HIV acquisition and transmission risks specific to transgender women (TGW) in SSA, recent studies indicate that HIV prevalence is also disproportionately high in this population across the region and even higher than that of cisgender MSM in certain settings [4–6]. Given the high burden of disease among sexual and gender minorities (SGM) in SSA, the lack of data characterizing HIV risk factors in these populations remains a critical barrier to achievement of the UNAIDS 95–95–95 targets in the region.

In addition to the transmission risk associated with condomless receptive anal sex without PrEP use [7], HIV vulnerability among SGM can be attributed to psychosocial factors, including SGM stigma [8–10]. Defined by sociologist Erving Goffman as the devaluation of an individual based on an attribute perceived by others as undesirable [11], stigma has consistently been identified as a barrier to HIV prevention among SGM [8, 9, 12]. MSM and TGW are highly stigmatized in many contexts in SSA, where the criminalization of male-to-male sexual contact incites and perpetuates discrimination and violence toward SGM and impedes HIV prevention efforts [4, 9, 13–16]. Previous work demonstrates that SGM who have experienced stigma or homonegative discrimination are more likely to be living with HIV in SSA [14, 17, 18], and SGM stigma has been associated with condomless anal sex among both MSM and TGW in the region [6, 19]. Associations between SGM stigma and fear of seeking healthcare services, as well as decreased knowledge of HIV transmission risks, have also been demonstrated among SGM in SSA [13, 20].

Recent work has highlighted the protective effect of social cohesion on SGM’s physical and mental health. Although social cohesion is not defined consistently throughout the literature, previous studies indicate that cohesion, or related concepts describing the closeness of one’s relationship to members of the SGM community, may reduce one’s risk of HIV [5, 17, 21]. Those with high social cohesion scores were significantly less likely to test positive for HIV in a sample of MSM and TGW in Côte d’Ivoire [5], while findings from a study of MSM in Eswatini showed that those who reported high social cohesion were approximately twice as likely to have received HIV testing in the past 12 months [21]. However, few studies have formally examined the potential moderating role of social cohesion in the relationship between stigma and HIV risk, and those that have offer mixed findings [22, 23].

In Zimbabwe, male-to-male sexual contact is criminalized and SGM stigma has been documented [24, 25], although few studies have addressed psychosocial determinants of health among Zimbabwean SGM. Zimbabwe has a generalized HIV epidemic, with a prevalence of 12.9% among adults aged 15 years and older [26]. While there are scant data describing the burden of HIV among SGM in Zimbabwe, we recently found that HIV prevalence was 17.1% and 28.0% among the 1,194 MSM and 344 TGW/genderqueer (GQ) individuals, respectively, in Harare and Bulawayo that participated in a biobehavioral survey (BBS) [27]. Given the high HIV prevalence and dearth of evidence regarding drivers of HIV vulnerability specific to Zimbabwean SGM, we examined SGM stigma as a possible risk factor and hypothesized that (1) SGM stigma is associated with HIV prevalence and risk factors among MSM and TGW/GQ individuals in Zimbabwe, and (2) social cohesion moderates the association between SGM stigma and HIV infection in this population. Characterizing the relationship between psychosocial factors and HIV vulnerability among MSM and TGW/GQ individuals in this context can support the development of comprehensive HIV prevention programs for Zimbabwean SGM and contribute to achievement of national HIV targets.

## Methods

### Study Design

#### Population, Sampling, and Survey Logistics

From March to July 2019, MSM and TGW/GQ individuals were recruited in Harare and Bulawayo, Zimbabwe, for a cross-sectional BBS. MSM and TGW/GQ individuals were eligible for BBS participation if they were born biologically male; engaged in anal or oral sex with a man in the previous 12 months; were aged 18 years or older; resided in Harare or Bulawayo in the previous month; and spoke English, Shona, or Ndebele.

Participants were recruited using respondent-driven sampling (RDS), a chain-referral recruitment technique developed to facilitate recruitment of hidden populations [28]. A total of 11 and eight seeds in Harare and Bulawayo, respectively, were purposively recruited by survey staff to reflect diverse socio-demographic characteristics. Using coded coupons, seeds were asked to recruit up to three eligible individuals from their social network, who then were asked to recruit from their own networks, creating recruitment chains until sample size targets were met. Participants were compensated United States Dollar (USD) 5 for each data collection visit and USD 5 for each peer (maximum of three) they successfully recruited for the survey. A total of 1538 participants (718 participants in Harare and 820 in Bulawayo) were enrolled.

#### Data Collection

After survey staff confirmed recruitment coupon validity and, subsequently, participant eligibility, all participants, or an authorized witness in cases where participants were illiterate, provided written, informed consent in their language of preference (English, Shona, or Ndebele). Participants completed an interviewer-administered structured questionnaire eliciting information on socio-demographic characteristics; sexual identity and history (including self-reported SGM group identity); HIV and sexually transmitted infection (STI)-related behaviors, knowledge, and attitudes; HIV prevention uptake; substance use; engagement with outreach and support programs; and experience with stigma and social cohesion. Questionnaires were translated from English to Shona and Ndebele using forward–backward translation to ensure consistency of meaning and were administered in the participant’s language of preference. After interview completion, consenting participants—regardless of self-reported HIV status—received on-site rapid HIV testing by trained nurses using an adaptation of the Zimbabwe National HIV Testing Algorithm [29]. Alere™ HIV Combo (Abbott Diagnostics Scarborough, Inc., ME, USA) was used as the first antibody test, in lieu of Determine or Standard Q, as it detects acute HIV infections better than other tests [30]. Chembio HIV 1/2 STAT-PAK™ (Chembio Diagnostic Systems, Inc., Hauppauge, NY, USA) and Insti HIV-1/HIV-2 Antibody Test (bioLytical Laboratories, BC, Canada) were used as second and third tests. Participants who tested positive for HIV received on-site CD4 and HIV recency testing and were referred to SGM-friendly facilities for care.

All procedures were approved by the Columbia University Irving Medical Center Institutional Review Board and the Medical Research Council of Zimbabwe. The protocol was also reviewed in accordance with the U.S. Centers for Disease Control and Prevention (CDC) human research protection procedures and was determined to be research, but CDC investigators did not interact with human subjects or have access to identifiable data or specimens for research purposes. Further information describing the survey methods have been published previously [31, 32].

### Key Measures

#### SGM Stigma and Social Cohesion

Items addressing stigma and social cohesion included in the questionnaire were adapted from the WHO Global HIV Strategic Information Working Group Biobehavioural Survey Guidelines for Populations at Risk for HIV [33] (Table 1). Participants were asked if they had faced nine different acts of stigmatization in workplace, community, or healthcare settings due to their SGM status (1 = no, 2 = yes, in the last 6 months, 3 = yes, but not in the last 6 months). Stigma variables were binarized (1 = yes, 0 = no) due to low frequencies. Social cohesion scores were derived from six survey items addressing trust, support, and solidarity within SGM communities, to which participants were asked to rate their agreement based on a five-point, Likert-type scale.

**Table 1:** SGM stigma and social cohesion construct measurement among MSM and TGW/GQ individuals, Zimbabwe, 2019

Scale	n (%)	Median (IQR)	Cronbach’s alpha
*Stigma*		1 (0–2)	0.62
Have you ever been arrested because you have sex with men?	71 (4.9)		
Have your friends or family left you or have you been rejected by them because you have sex with men?	211 (14.6)		
Have you been terminated from a job because you have sex with men?	40 (2.8)		
Have you been denied a job because you have sex with men?	48 (3.3)		
Have you ever been blackmailed by someone because you have sex with men?	255 (15.6)		
Have you ever been treated unfairly or denied healthcare because you have sex with men?	49 (3.40)		
Have you ever avoided seeking healthcare services because you were worried someone may learn you have sex with men?	265 (18.4)		
When you seek health care, do you feel you need to hide that you have sex with men?	483 (33.5)		
Have you ever suffered any physical, sexual, or verbal harassment or abuse because you have sex with men?	433 (30.1)		
*Response options: 1* = *Yes, 0* = *No*^a^			

Scale		Median (IQR)	Cronbach’s alpha
*Social cohesion*		16 (12–18)	0.83
You can count on other MSM or TGW/GQ individuals if you need to borrow money		4 (2–5)	
You can count on other MSM or TGW/GQ individuals to accompany you to the doctor or hospital		4 (2–5)	
You can count on other MSM or TGW/GQ individuals if you need to talk about your problems		4 (3–5)	
You can count on other MSM or TGW/GQ individuals if you need a place to stay		4 (4–5)	
*Response options: 1* = *Strongly disagree, 2* = *Disagree, 3* = *Neutral, 4* = *Agree, 5* = *Strongly agree*			

^a^Original questionnaire included 3 response options for stigma items (1 = No, 2 = Yes, in the last 6 months, 3 = Yes, not in the last 6 months); items were recoded as binary (1 = Yes, 0 = No) due to low frequencies

Exploratory factor analysis (EFA) with orthogonal rotation was conducted for both stigma and social cohesion to assess unidimensionality, which was confirmed for both scales. The following two social cohesion items were dropped due to low factor loadings, which indicate correlation between the variable and underlying factor: (1) In the past 6 months, have you negotiated with or stood up against a non-MSM in order to help a fellow MSM or TGW/GQ?; (2) During the past 6 months, how often have you gone to a support group for gay men, MSM, or TGW/GQ? The remaining four social cohesion items exhibited high internal consistency on the scale (Cronbach’s alpha = 0.83), while the nine stigma items exhibited adequate internal consistency (Cronbach’s alpha = 0.62). Stigma and social cohesion scores were quantified by summing responses across the respective survey questions. Scores were dichotomized at the median for both stigma (median = 1) and social cohesion (median = 16), yielding final binary measures for both constructs. Dichotomization of scales has been used previously to quantify stigma and social support measures to ease interpretation, particularly when a skewed distribution is observed [34, 35].

EFA and scale dichotomization was conducted within the combined sample, as a sensitivity analysis demonstrated that performing these methods within the Harare and Bulawayo samples separately did not yield analytic differences in the results of the EFA or our final models (data not shown).

#### HIV Prevalence and Associated Behaviors

HIV test result determined during the survey was examined as the primary outcome variable. HIV testing behavior, condom use, and comprehensive HIV knowledge were examined as secondary outcomes. HIV testing behavior history was assessed by asking participants if they had ever received HIV testing prior to the survey. Condom use at last sex with main male partners, defined as the male partner with whom the participant had sex with the most in the past 6 months, and casual male partners was measured with a binary yes/no response. Comprehensive HIV knowledge was defined as correctly answering five questions about HIV transmission risk, aligned with the UNAIDS definition [36].

### Analysis

All analyses were conducted using SAS version 9.4 (SAS Institute, Cary, NC, USA). A total of 70 participants who answered “don’t know” or “refused to answer” to stigma or social cohesion variables, or when asked if they had receptive or insertive anal sex at last sex with their main male partner, were excluded from analyses. Data were not imputed given that these cases represented < 5% of the analytic sample [37]. Harare and Bulawayo represented discrete analytic samples and, therefore, were analyzed separately. Within each city, TGW and GQ individuals were combined into a single group based on guidance from local key populations organizations and to ensure model convergence given the small number of non-MSM participants. All estimates are RDS-unadjusted as the sample did not reach convergence for HIV status; therefore, analyses were unweighted and did not account for sampling design.

Chi-square tests, or Fisher’s exact tests in cases where 25% or more cell sizes were 5 or below, were used to assess associations between socio-demographic variables and levels of stigma and social cohesion. Relative risk regression was used to measure associations between stigma and the outcome variables of interest. This method was employed as it has been shown to provide more accurate measures of association given a cross-sectional design and non-rare outcome variables [38, 39], which was the case in our analysis, and effect measures are interpreted as prevalence ratios. To overcome the convergence issues associated with traditional log-binomial relative risk regression models [40], a Poisson distribution with robust error variance was used with participants’ coupon number serving as the subject ID. Separate bivariate regression models were fit to assess the main effect of SGM stigma on the primary and secondary outcomes of interest, including HIV status, HIV testing behavior, comprehensive HIV knowledge, and condom use at last sex with both main and casual male partners. Multivariable models were fit to examine the effect of SGM stigma on the outcome variables while adjusting for age and SGM group, which were identified as potential confounders based on causal theory and descriptive statistics. Although marital status and education were also identified as potential confounders, they were ultimately excluded as co-variates given that they were not significant in any models and did not provide sufficient cell sizes for each stratum. Finally, in order to examine social cohesion as a potential moderator, social cohesion and its interaction with SGM stigma were added to any multivariable regression models in which SGM stigma was significantly associated with the outcome in adjusted analyses. All models were additionally fit to a sample restricted to MSM in a post-hoc analysis performed to examine the potential for differences between SGM groups.

## Results

### Participant Characteristics

Of the 1538 MSM and TGW/GQ individuals in Harare (n = 718) and Bulawayo (n = 820) who were enrolled in the BBS and interviewed using the questionnaire, 1511 (98.2%) consented to HIV testing. In Chi-square and Fisher’s exact tests, biomarker testing consent was associated with income (χ^2^ = 16.71, p = 0.0002), education (χ^2^ = 9.46, p = 0.01), and marital status (Fisher’s exact p = 0.01). Biomarker testing consent was also associated with social cohesion, in that participants with low social cohesion were significantly less likely to consent to HIV testing compared to those with high social cohesion (χ^2^ = 178.18, p < 0.0001). Participants who did not consent to HIV testing were excluded from the analytic sample.

The final analytic sample included 662 SGM from Harare and 779 from Bulawayo. Overall, the most commonly reported acts of stigma were feeling it necessary to hide that one has sex with men while seeking healthcare (33.5%); having experienced physical, sexual, or verbal harassment due to having sex with men (30.1%); and having avoided seeking healthcare services due to concerns about disclosure of one’s SGM status (18.4%).

Socio-demographic characteristics of participants stratified by city and levels of SGM stigma are shown in Table 2. The median age of participants was 24 years (interquartile range [IQR] 21–29) in Harare and 26 years (IQR 22–34) in Bulawayo, and the sample was predominantly MSM (59.5% in Harare and 93.1% in Bulawayo). Most participants in Harare (69.6%) and Bulawayo (72.9%) had attended secondary school. Approximately half (54.4% in Harare and 43.8% in Bulawayo) were currently employed and median income earned in the previous month was USD 100 (IQR 50–150) in Harare and USD 80 (IQR 50–150) in Bulawayo. The majority of participants had never been married (81.7% in Harare and 80.8% in Bulawayo) and were not currently living with a sexual partner (86.4% in Harare and 83.3% in Bulawayo). In sum, the demographic profiles of participants were similar in the two cities.

**Table 2:** Socio-demographic characteristics of MSM and TGW/GQ individuals stratified by levels of SGM stigma, Zimbabwe, 2019

	Harare (n = 662)					Bulawayo (n = 779)				
	SGM stigma					SGM stigma				
	Total	High	Low	*x*^2^	p-value	Total	High	Low	*x*^2^	p-value
	n (%)	n (%)	n (%)			n (%)	n (%)	n (%)		
Total sample	662	456 (68.9)	206 (31.1)			779	509 (65.3)	270 (34.7)		
Age				1.14	0.77				8.72	0.03*
18–24	352 (53.2)	243 (53.3)	109 (52.9)			317 (40.7)	212 (41.6)	105 (38.9)		
25–34	232 (35.0)	163 (35.7)	69 (33.5)			282 (36.2)	194 (38.1)	88 (32.6)		
35–44	60 (9.1)	39 (8.6)	21 (10.2)			119 (15.3)	72 (14.2)	47 (17.4)		
45 or older	18 (2.7)	11 (2.4)	7 (3.4)			61 (7.8)	31 (6.1)	30 (11.1)		
Median (IQR)	24 (21–29)					26 (22–34)				
SGM group				3.16	0.08				21.70	< 0.0001*
MSM	394 (59.5)	261 (57.2)	133 (64.6)			725 (93.1)	458 (90.0)	267 (98.9)		
TGW/GQ	268 (40.5)	195 (42.8)	73 (35.4)			54 (6.9)	51 (10.0)	3 (1.1)		
Race				N/A	0.32^a^				0.33	0.57
Black African	658 (99.4)	452 (99.1)	206 (100.0)			755 (96.9)	492 (96.7)	263 (97.4)		
Other	4 (0.6)	4 (0.9)	0			24 (3.1)	17 (3.3)	7 (2.6)		
Nationality				N/A	0.67^a^				N/A	0.39^a^
Zimbabwean	656 (99.1)	451 (98.9)	205 (99.5)			766 (98.3)	502 (98.6)	264 (97.8)		
Other	6 (0.9)	5 (1.1)	1 (0.5)			13 (1.7)	7 (1.4)	6 (2.2)		
Highest education attended				6.09	0.04*				8.54	0.01*
None or primary	9 (1.4)	9 (2.0)	0			70 (9.0)	43 (8.5)	27 (10.0)		
Secondary	461 (69.6)	308 (67.5)	153 (74.3)			568 (72.9)	359 (70.5)	209 (77.4)		
Tertiary or vocational	192 (29.0)	139 (30.5)	53 (25.7)			141 (18.1)	107 (21.0)	34 (12.6)		
Literacy				N/A	0.53^a^				0.33	0.57
Can read and write	660 (99.7)	455 (99.8)	205 (99.5)			762 (97.8)	499 (98.0)	263 (97.4)		
Cannot read or write or can read only	2 (0.3)	1 (0.2)	1 (0.5)			17 (2.2)	10 (2.0)	7 (2.6)		
Employment status				N/A	0.39^a^				N/A	0.68^a^
Unemployed	209 (31.6)	135 (29.6)	74 (35.9)			337 (43.3)	214 (42.0)	123 (45.6)		
Employed	360 (54.4)	255 (55.9)	105 (51.0)			341 (43.8)	225 (44.2)	116 (43.0)		
Full-time student	92 (13.9)	65 (14.3)	27 (13.1)			98 (12.3)	67 (13.2)	29 (10.7)		
Retired or other	1 (0.1)	1 (0.2)	0			5 (0.6)	3 (0.6)	2 (0.7)		
Income earned last month (USD)				1.75	0.42				1.01	0.60
0–149	254 (38.4)	182 (39.9)	72 (35.0)			256 (32.9)	165 (32.4)	91 (33.7)		
150 or greater	107 (16.1)	74 (16.2)	33 (16.0)			84 (10.8)	59 (11.6)	25 (9.3)		
No answer, student, or unemployed	301 (45.5)	200 (43.9)	101 (49.0)			439 (56.3)	285 (56.0)	154 (57.0)		
Median (IQR)	100 (50–150)					80 (50–150)				
Marital status				4.35	0.11				10.81	0.005*
Single, never married	541 (81.7)	382 (83.8)	159 (77.2)			629 (80.8)	428 (84.1)	201 (74.4)		
Married or cohabiting	51 (7.7)	30 (6.6)	21 (10.2)			47 (6.0)	24 (4.7)	23 (8.5)		
Separated, divorced, or widowed	70 (10.6)	44 (9.6)	26 (12.6)			103 (13.2)	57 (11.2)	46 (17.1)		
Currently living with a sexual partner				1.50	0.22				0.15	0.69
Yes	90 (13.6)	67 (14.7)	23 (11.2)			130 (16.7)	83 (16.3)	47 (17.4)		
No	572 (86.4)	389 (85.3)	183 (88.8)			649 (83.3)	429 (83.7)	223 (82.6)		
Regular place to sleep at night				N/A	0.45^a^				N/A	0.76^a^
Yes	654 (98.8)	449 (98.5)	205 (99.5)			767 (98.5)	502 (98.6)	265 (98.2)		
No	8 (1.2)	7 (1.5)	1 (0.5)			12 (1.5)	7 (1.4)	5 (1.8)		
Shelter type				N/A	0.71^a^				N/A	0.10^a^
House or apartment	654 (98.8)	451 (98.9)	203 (98.5)			769 (98.7)	505 (99.2)	264 (97.8)		
Other	8 (1.2)	5 (1.1)	3 (1.5)			10 (1.3)	4 (0.8)	6 (2.2)		
Condom use at last sex with main male partner				4.10	0.04*				0.47	0.49
Yes	243 (36.7)	179 (39.2)	64 (31.1)			236 (30.3)	150 (29.5)	86 (31.9)		
No	419 (63.3)	277 (60.8)	142 (68.9)			543 (69.7)	359 (70.5)	184 (68.1)		
Condom use at last sex with casual male partner^b^				0.21	0.65				0.32	0.57
Yes	72 (20.4)	50 (19.8)	22 (22.0)			66 (20.9)	46 (21.8)	20 (19.1)		
No	280 (79.6)	202 (80.2)	78 (78.0)			250 (79.1)	165 (78.2)	85 (80.9)		

*Significant association (p < 0.05)

^a^Fisher’s exact p-value used due to ≥ 25% of cell counts being 5 or below

^b^Among those with ≥ 1 casual male partner in last 6 months (Harare: n = 352, Bulawayo: n = 316)

Prevalence of ever experiencing SGM stigma was 68.9% in Harare and 65.3% in Bulawayo. In Harare, SGM stigma was associated with education (χ^2^ = 6.09, p = 0.04) and condom use at last sex with a main male partner (χ^2^ = 4.10, p = 0.04). In Bulawayo, SGM stigma was associated with age (χ^2^ = 8.72, p = 0.03), SGM group (χ^2^ = 21.70, p < 0.0001), education (χ^2^ = 8.54, p = 0.01), and marital status (χ^2^ = 10.81, p = 0.005).

### SGM Stigma and HIV Risk

Results of relative risk regression analyses examining the relationship between SGM stigma and HIV-related outcomes are presented in Table 3. In bivariate analyses, SGM stigma was significantly associated with testing HIV-positive in Harare (prevalence ratio [PR] = 1.87, 95% confidence interval [CI] = 1.28–2.73, Z = 3.25, p = 0.001) but not in Bulawayo (PR = 1.30, 95% CI = 0.98–1.72, Z = 1.82, p = 0.07). However, after adjusting for age and SGM group, there was a significant association between SGM stigma and testing HIV-positive in both Harare [adjusted PR (aPR) = 1.82, 95% CI = 1.27–2.62, Z = 3.22, p = 0.001] and Bulawayo (aPR = 1.51, 95% CI = 1.15–2.00, Z = 2.92, p = 0.004). No significant associations were found between SGM stigma and ever testing for HIV, comprehensive HIV knowledge, or condom use at last sex with a main or casual male partner in unadjusted or adjusted analyses in either city (Table 3). Restricting the sample to MSM yielded similar results (data not shown).

**Table 3:** Associations between SGM stigma and HIV-related outcomes among MSM and TGW/GQ individuals by city, Zimbabwe, 2019

	SGM stigma (Yes vs. no)											
	Harare						Bulawayo					
	Unadjusted PR (95% CI)	Z	p-value	Adjusted PR (95% CI)^a^	Z	p-value	Unadjusted PR (95% CI)	Z	p-value	Adjusted PR (95% CI)^a^	Z	p-value
Outcome variable												
HIV positive	1.87 (1.28–2.73)*	3.25	0.001	1.82 (1.27–2.62)*	3.22	0.001	1.30 (0.98–1.72)	1.82	0.07	1.51 (1.15–2.00)*	2.92	0.004
Ever tested for HIV	0.98 (0.92–1.03)	− 0.84	0.40	0.98 (0.92–1.04)	− 0.78	0.44	1.04 (0.97–1.12)	1.15	0.25	1.03 (0.96–1.11)	0.79	0.43
Comprehensive HIV knowledge	0.97 (0.90–1.05)	− 0.66	0.51	0.98 (0.90–1.06)	− 0.54	0.59	1.03 (0.92–1.15)	0.54	0.59	1.07 (0.96–1.19)	1.16	0.25
Did not use condom at last sex (main partner)	1.26 (1.0–1.59)	1.97	0.05	1.24 (0.99–1.57)	1.83	0.07	0.92 (0.74–1.15)	− 0.69	0.49	0.93 (0.74–1.16)	− 0.65	0.51
Did not use condom at last sex (casual partner)^b^	0.90 (0.58–1.41)	− 0.46	0.65	0.90 (0.58–1.40)	− 0.48	0.63	1.14 (0.71–1.83)	0.56	0.57	1.43 (0.88–2.32)	1.43	0.15

*Significant association (p < 0.05)

^a^Separate multivariable models adjusted for age (continuous) and SGM group (1 = MSM [reference], 2 = TGW/GQ)

^b^Model restricted to those with ≥ 1 casual male partner in last 6 months (Harare: n = 352, Bulawayo: n = 316)

### Social Cohesion

The prevalence of high social cohesion was 33.2% in Harare and 65.3% in Bulawayo. Socio-demographic characteristics of the sample stratified by levels of social cohesion and city are shown in Table 4. In Harare, high social cohesion was associated with marital status (χ^2^ = 7.74, p = 0.02) and condom use at last sex with a casual male partner (χ^2^ = 4.72, p = 0.03). In Bulawayo, high social cohesion was associated with SGM group (χ^2^ = 4.66, p = 0.03) and education (χ^2^ = 9.21, p = 0.01). There was no evidence that social cohesion moderated the association between SGM stigma and testing HIV-positive in either city (Table 5).

**Table 4:** Socio-demographic characteristics of MSM and TGW/GQ individuals stratified by levels of social cohesion, Zimbabwe, 2019

	Harare (n = 662)					Bulawayo (n = 779)				
	Social cohesion					Social cohesion				
	Total	High	Low	*x*^2^	p-value	Total	High	Low	*x*^2^	p-value
	n (%)	n (%)	n (%)			n (%)	n (%)	n (%)		
Total sample	662	220 (33.2)	442 (66.8)			779	509 (65.3)	270 (34.7)		
Age				0.72	0.87				2.09	0.55
18–24	352 (53.2)	122 (55.5)	230 (52.0)			317 (40.7)	200 (39.3)	117 (43.3)		
25–34	232 (35.0)	73 (33.2)	159 (36.0)			282 (36.2)	184 (36.1)	98 (36.3)		
35–44	60 (9.1)	19 (8.6)	41 (9.3)			119 (15.3)	82 (16.1)	37 (13.7)		
45 or older	18 (2.7)	6 (2.7)	12 (2.7)			61 (7.8)	43 (8.5)	18 (6.7)		
Median (IQR)	24 (21–29)					26 (22–34)				
SGM group				0.03	0.86				4.66	0.03*
MSM	394 (59.5)	132 (60.0)	262 (59.3)			725 (93.1)	481 (94.5)	244 (90.4)		
TGW/GQ	268 (40.5)	88 (40.0)	180 (40.7)			54 (6.9)	28 (5.5)	26 (9.6)		
Race				N/A	0.60^a^				1.37	0.24
Black African	658 (99.4)	218 (99.1)	440 (99.5)			755 (96.9)	496 (97.4)	259 (95.9)		
Other	4 (0.60)	2 (0.9)	2 (0.5)			24 (3.1)	13 (2.6)	11 (4.1)		
Nationality				N/A	1.0^a^				N/A	0.77^a^
Zimbabwean	656 (99.1)	218 (99.1)	438 (99.1)			766 (98.3)	501 (98.4)	265 (98.1)		
Other	6 (0.9)	2 (0.9)	4 (0.9)			13 (1.7)	8 (1.6)	5 (1.9)		
Highest education attended				0.78	0.68				9.21	0.01*
None or primary	9 (1.4)	2 (0.9)	7 (1.6)			70 (9.0)	52 (10.2)	18 (6.7)		
Secondary	461 (69.6)	151 (68.6)	310 (70.1)			568 (72.9)	379 (74.5)	189 (70.0)		
Tertiary or vocational	192 (29.0)	67 (30.5)	125 (28.3)			141 (18.1)	78 (15.3)	63 (23.3)		
Literacy				N/A	1.0^a^				0.21	0.65
Can read and write	660 (99.7)	220 (100.0)	440 (99.5)			762 (97.8)	497 (97.6)	265 (98.1)		
Cannot read or write or can read only	2 (0.30)	0	2 (0.5)			17 (2.2)	12 (2.4)	5 (1.9)		
Employment status				N/A	0.81^a^				N/A	0.18^a^
Unemployed	209 (31.6)	69 (31.4)	140 (31.7)			337 (43.3)	223 (43.8)	114 (42.2)		
Employed	360 (54.4)	117 (53.2)	243 (55.0)			341 (43.8)	229 (45.0)	112 (41.5)		
Full-time student	92 (13.9)	34 (15.4)	58 (13.1)			96 (12.3)	55 (10.8)	41 (15.2)		
Retired or other	1 (0.1)	0	1 (0.2)			5 (0.6)	2 (0.4)	3 (1.1)		
Income earned last month (USD)				2.91	0.23				0.90	0.64
0–149	254 (38.4)	89 (40.5)	165 (37.3)			256 (32.9)	173 (34.0)	83 (30.7)		
150 or greater	107 (16.1)	28 (12.7)	79 (17.9)			84 (10.8)	53 (10.4)	31 (11.5)		
No answer, student, or unemployed	301 (45.5)	103 (46.8)	198 (44.8)			439 (56.3)	283 (55.6)	156 (57.8)		
Median (IQR)	100 (50–150)					80 (50–150)				
Marital status				7.74	0.02*				0.34	0.84
Single, never married	541 (81.7)	182 (82.7)	359 (81.2)			629 (80.8)	408 (80.1)	221 (81.9)		
Married or cohabiting	51 (7.7)	9 (4.1)	42 (9.5)			47 (6.0)	32 (6.3)	15 (5.5)		
Separated, divorced, or widowed	70 (10.6)	29 (13.2)	41 (9.3)			103 (13.2)	69 (13.6)	34 (12.6)		
Currently living with a sexual partner				0.49	0.48				0.0001	0.99
Yes	90 (13.6)	27 (12.3)	63 (14.2)			130 (16.7)	85 (16.7)	45 (16.7)		
No	572 (86.4)	193 (87.7)	379 (85.8)			649 (83.3)	424 (83.3)	225 (83.3)		
Regular place to sleep at night				N/A	0.73^a^				N/A	0.76^a^
Yes	654 (98.8)	219 (99.5)	435 (98.4)			767 (98.5)	502 (98.6)	265 (98.1)		
No	8 (1.2)	1 (0.5)	7 (1.6)			12 (1.5)	7 (1.4)	5 (1.9)		
Shelter type				N/A	0.28^a^				N/A	0.75^a^
House or apartment	654 (98.8)	219 (99.5)	435 (98.4)			769 (98.7)	503 (98.8)	266 (98.5)		
Other	8 (1.2)	1 (0.5)	7 (1.6)			10 (1.3)	6 (1.2)	4 (1.5)		
Condom use at last sex with main male partner				0.05	0.83				0.47	0.49
Yes	243 (36.7)	82 (37.3)	161 (36.4)			543 (69.7)	150 (29.5)	86 (31.9)		
No	419 (63.3)	138 (62.7)	281 (63.6)			236 (30.3)	359 (70.5)	184 (68.1)		
Condom use at last sex with casual male partner^b^				4.72	0.03*				0.32	0.57
Yes	72 (20.5)	33 (26.8)	39 (17.0)			66 (20.9)	46 (21.8)	20 (19.1)		
No	280 (79.5)	90 (73.2)	190 (83.0)			250 (79.1)	165 (78.2)	85 (80.9)		

*Significant association (p < 0.05)

^a^Fisher’s exact p-value used due to ≥ 25% of cell counts being 5 or below

^b^Among those with ≥ 1 casual male partner in last 6 months (Harare: n = 352, Bulawayo: n = 316)

**Table 5:** Interaction between SGM stigma and social cohesion among MSM and TGW/GQ individuals by city, Zimbabwe, 2019

	Social cohesion and stigma interaction					
	Harare			Bulawayo		
	Adjusted PR (95% CI)^a^	Z	p-value	Adjusted PR (95% CI)^a^	Z	p-value
Outcome variable						
HIV positive	0.72 (0.33–1.58)	− 0.81	0.42	0.86 (0.49–1.52)	− 0.51	0.61

^a^Separate multivariable models adjusted for age (continuous) and SGM group (1 = MSM [reference], 2 = TGW/GQ)

## Discussion

This is the first study characterizing associations between SGM stigma, social cohesion, and HIV risk among SGM in Zimbabwe. We demonstrate one and a half to nearly two-fold times higher HIV prevalence among MSM and TGW/GQ individuals in our sample who had experienced a higher level of SGM stigma. This is consistent with previous research among MSM in South Africa, Lesotho, and Uganda [14, 17, 18] and suggests that stigma may contribute to the burden of HIV among SGM in Zimbabwe. Data describing psychosocial factors associated with HIV among Zimbabwean SGM are critically important and equally difficult to obtain given the criminalized and heavily stigmatized nature of non-heterosexual relationships. Our study therefore provides meaningful findings that may inform HIV programming for SGM and contribute to achievement of national HIV targets.

Social cohesion did not moderate the association between SGM stigma and HIV test result. This was contrary to our hypothesis and previous literature [5, 21], although few studies have formally evaluated the moderating role of social cohesion in the relationship between SGM stigma and HIV risk. Additionally, we found no associations between SGM stigma and comprehensive HIV knowledge, HIV testing behavior, or condomless anal sex. Similar to our findings, previous work has also identified a relationship between SGM stigma and HIV prevalence while finding non-significant associations between SGM stigma and specific HIV risk factors [17]. These results likely reflect the challenges associated with measuring sexual activity and behaviors [41, 42]. Reliable and valid measures of condom use are particularly challenging to obtain, as self-reported information is subject to bias and it is unclear which temporal period best captures one’s condom use behavior. Additionally, the relationship between stigma and HIV status is complex, as SGM often experience multiple forms of stigma as well as intersecting stigmas related to their membership in more than one marginalized group, including sex workers and people living with HIV (PLHIV) [43, 44]. Further research should examine the effects of discrete forms of stigma as well as possible interactions between stigmatized identities to further elucidate the relationship between SGM stigma and HIV risk.

Our study and analysis have several limitations. Stigma theory supports the plausibility of a temporal relationship between SGM stigma and HIV risk [45, 46], but our cross-sectional survey design precluded determination of the direction of this association. Additionally, while the stigma items in our survey were informed by the WHO Biobehavioural Survey Guidelines for Populations at Risk for HIV [33], the stigma scale showed only adequate internal consistency among our population of interest (Cronbach’s alpha = 0.62). Future work may benefit from further scale validation in order to develop a stigma scale with higher internal consistency among MSM and TGW/GQ individuals in Zimbabwe. Furthermore, our Harare and Bulawayo populations were treated as convenience samples due to our inability to use RDS weights to adjust for non-random recruitment patterns. Therefore, the results of this analysis do not represent population estimates and may not be generalizable to all MSM and TGW/GQ individuals in Harare and Bulawayo. Our analytic sample also combined MSM and TGW/GQ participants based on country context and to ensure convergence on all models, although prior literature suggests that SGM sub-groups may experience stigma and social cohesion differently [5, 6]. While our results did not differ when restricting the sample to MSM, future research that disaggregates SGM groups is needed, given a sufficient sample size. Despite these limitations, ours is the first study to examine stigma and social cohesion as possible psychosocial determinants of HIV prevalence among SGM in Zimbabwe. Strengths of our study include the use of RDS to recruit hard-to-reach SGM who may have limited exposure to HIV programming or may choose not to disclose male-to-male sexual practices during the Zimbabwe Population-based HIV Impact Assessment (ZIMPHIA) or other household-based methodologies traditionally used for survey data collection in the country and region.

The greater HIV prevalence among SGM who had experienced stigma in our study is an important finding, underscoring the 2014 Zimbabwe Stigma Index Report’s call for interventions against SGM stigma [47] and demonstrating the potential benefit of integrating stigma-mitigation efforts into HIV prevention and care programs. Extensive healthcare-related stigma toward MSM has been demonstrated in previous work, including a study exploring stigma among the HTPN 075 cohort of MSM and TGW in Kenya, Malawi, and South Africa, in which nearly half of participants reported one or more healthcare-related stigma experience [48]. Stigma by healthcare providers is an important barrier to HIV prevention, given that MSM and TGW may avoid seeking services if they have experienced or anticipate stigma in healthcare settings. Furthermore, healthcare-related stigma toward MSM and TGW may hinder progress toward national and global goals such as the 95–95–95 HIV targets, as it has been demonstrated that MSM who experienced stigma are more likely to delay enrollment in HIV care services until they are critically ill [8]. Successful stigma-mitigation programs in the region include an integrated stigma-mitigation intervention in Senegal with community, clinical, and peer-focused components that significantly decreased fear of seeking healthcare services among MSM [49] as well as a MSM-sensitization program for Kenyan healthcare providers that improved MSM engagement in health services [50]. Similar initiatives may be considered for Zimbabwean SGM, given that avoidance of healthcare services and concealment of SGM status while seeking healthcare were two of the most commonly reported acts of stigma in our study. Similarly, findings from a qualitative study describing the experience of MSM accessing HIV services in Bulawayo, Zimbabwe indicated that MSM who disclosed their sexual orientation often experienced stigmatization by healthcare providers, leading to fear of seeking services [51].

While mitigating healthcare-related stigma is critical, as Mutanga and Moen describe in their 2019 qualitative analysis of sexuality-related stigma among gay and bisexual men in Zimbabwe, reducing stigma among SGMs in this context requires multifaceted interventions targeting stigma at the individual, community, and government levels [25]. A combination intervention focused on SGM’s access to healthcare, social perceptions of SGM, and the legal context surrounding non-heterosexual relationships in Zimbabwe will likely be more effective than either a universal or unidimensional approach. Integrating comprehensive stigma-mitigation interventions into HIV prevention and care programs is critical for ensuring progress towards HIV elimination in Zimbabwe.

## Data Availability

A de-identified dataset may be made available upon reasonable request and obtainment of all necessary institutional approvals.

## Code Availability

All analyses were conducted in SAS version 9.4 (SAS Institute, Cary, NC, USA). SAS code may be made available upon request.
